# First Successful Regeneration of a Structurally Native‐Like Colon Using a Bioabsorbable Polymer Sheet

**DOI:** 10.1002/ags3.70137

**Published:** 2025-11-21

**Authors:** Junpei Takashima, Mitsuo Miyazawa, Masayasu Aikawa, Daisuke Fujimoto, Hirotoshi Kobayashi

**Affiliations:** ^1^ Department of Surgery Teikyo University School of Medicine, Mizonokuchi Hospital Kawasaki Japan; ^2^ Department of Preventive Medicine International University of Health and Welfare Chiba Japan; ^3^ Department of Gastroenterological Surgery Saitama Medical University International Medical Center Saitama Japan

**Keywords:** animals, bioabsorbable materials, colon, intestinal perforation, tissue regeneration

## Abstract

**Background and Aim:**

In cases of colonic perforation, closure of the defect is the least invasive treatment; however, no dedicated tool has been developed for this purpose, and many cases require stoma creation. This study investigated whether a bioabsorbable polymer sheet (BAPS) can effectively close colonic perforation and induce colonic regeneration at the defect site.

**Methods:**

Ten mixed‐breed pigs were used. An artificial colon was created using a BAPS composed of a copolymer reinforced with polyglycolic acid fibers. A circular 2‐cm defect was created on the anterior sigmoid colon wall, and the BAPS was patched over the defect. Blood sampling and body weight assessments were performed at 1, 4, and 8 weeks after transplantation. Furthermore, the transplanted site was excised for macroscopic and histological evaluation at 4 and 8 weeks.

**Results:**

All pigs survived without complications. White blood cell counts exhibited a transient increase at 1 week but normalized rapidly. Body weight increased consistently. At 4 weeks, the BAPS was no longer observed at the transplanted site, and ulcer formation was noted. Histologically, several immature cells accumulated on the epithelial surface. At 8 weeks, the ulcers had reduced in size, and the site was indistinguishable from the surrounding tissue. Histological examination revealed regenerated mucosa and muscularis mucosae resembling native colonic tissue with partial smooth muscle regeneration. The size of the transplanted site was reduced by approximately 26%.

**Conclusions:**

The findings suggest that a BAPS can successfully close colonic perforations and regenerate colonic tissue at the defect site.

## Graphical abstract

This study demonstrated that a bioabsorbable polymer sheet (BAPS) can regenerate approximately half the circumference of the colon with native‐like structure and function in an experimental setting. As the model involved immediate closure after perforation and did not reproduce peritonitis, caution is required when considering clinical applicability. Nevertheless, BAPS may represent a promising option for minimally invasive repair of colonic perforations, particularly when applied endoscopically in cases such as iatrogenic perforation after ESD.

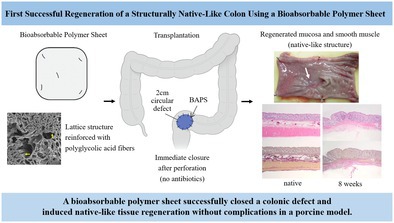

## 1 Introduction

The colon is a harsh environment where numerous bacteria reside within the intestinal lumen. When colonic perforation occurs because of iatrogenic causes, such as endoscopic procedures, diverticulitis, and colorectal cancer, peritonitis often develops, necessitating surgical intervention. In such cases, the surrounding tissue is subjected to severe inflammation, thereby making colonic wall repair challenging.

The incidence of colonic diverticulosis has been increasing recently. In cases of perforation, emergency surgery is often required, frequently leading to the creation of a stoma [1, 2]. Furthermore, the use of endoscopic submucosal dissection (ESD) for early‐stage colorectal cancer has been increasing. However, ESD is associated with a high risk of perforation, particularly in the colon [3, 4], with reports indicating that approximately 15% of perforation cases require stoma creation [5]. In addition, perforation occurs in 3%–10% of colorectal cancer cases, and approximately half of the patients undergoing emergency surgery require a stoma [6, 7]. These findings emphasize that colonic perforation is not a rare condition, and primary closure is often challenging. Therefore, extensive colectomy, including perforated segment or stoma creation, is frequently necessary, posing a significant clinical challenge.

The creation of a stoma significantly reduces patients' quality of life and requires additional surgery for stoma closure, increasing healthcare costs. If a material that can directly cover the perforation site while promoting organ regeneration were available, minimally invasive treatment can be achieved while preserving function and eliminating the need for extensive colonic resection or stoma creation. This approach is ideal for reducing healthcare expenditures.

To date, various studies on organ regeneration using organoids, expanded polytetrafluoroethylene (ePTFE), and small intestinal submucosa (SIS) have been conducted [8, 9, 10]. However, none of these approaches were ideal for clinical application.

In this study, a bioabsorbable polymer (BAP) was developed based on recent advances in tissue engineering. We successfully used BAP technology to regenerate several organs, including the bile duct [11], esophageal wall [12], gastric wall [13], inferior vena cava [14], and portal vein [15]. In this study, we investigated whether an artificial colon constructed using a BAP could regenerate colonic tissue even in the presence of a certain degree of infection. We also aimed to develop a novel treatment approach for colonic perforation by directly closing the defect using an artificial colon.

## 2 Materials and Methods

### 2.1 The Artificial Colon

**FIGURE 1:**
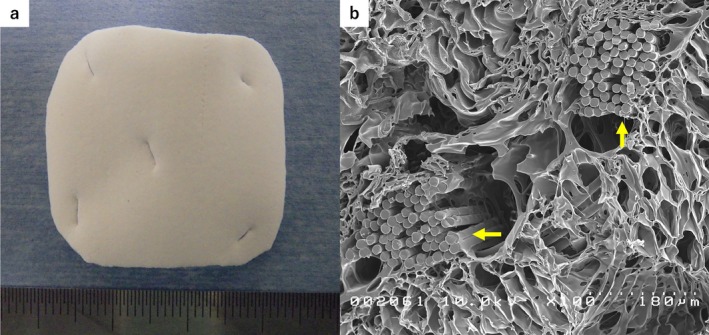
Artificial colon. (a) Bioabsorbable polymer sheet (BAPS). The BAPS was circular in shape measuring 2.5 × 2.5 cm. (b) Scanning electron microscope image. The BAPS is made of fibers comprising a copolymer (50% L‐lactic acid and 50% polycaprolactone) reinforced with polyglycolic acid fibers, and it has a lattice‐like structure (yellow arrows).

The artificial colon was constructed using a BAP sheet (BAPS) composed of poly(L‐lactic acid) and polycaprolactone (50:50) copolymer fibers reinforced with polyglycolic acid (PGA) fibers (Figure 1a). These fibers were arranged in a lattice structure, designed to resist tearing when sutured with a surgical needle or grasped with forceps (Figure 1b). Based on previous experience in organ regeneration experiments, the material was particularly designed for colonic wall regeneration. The sheet was engineered to be absorbed within approximately 6–8 weeks; it has a thickness of approximately 1 mm and an air porosity of < 95%.

### 2.2 Animal Surgery

**FIGURE 2:**
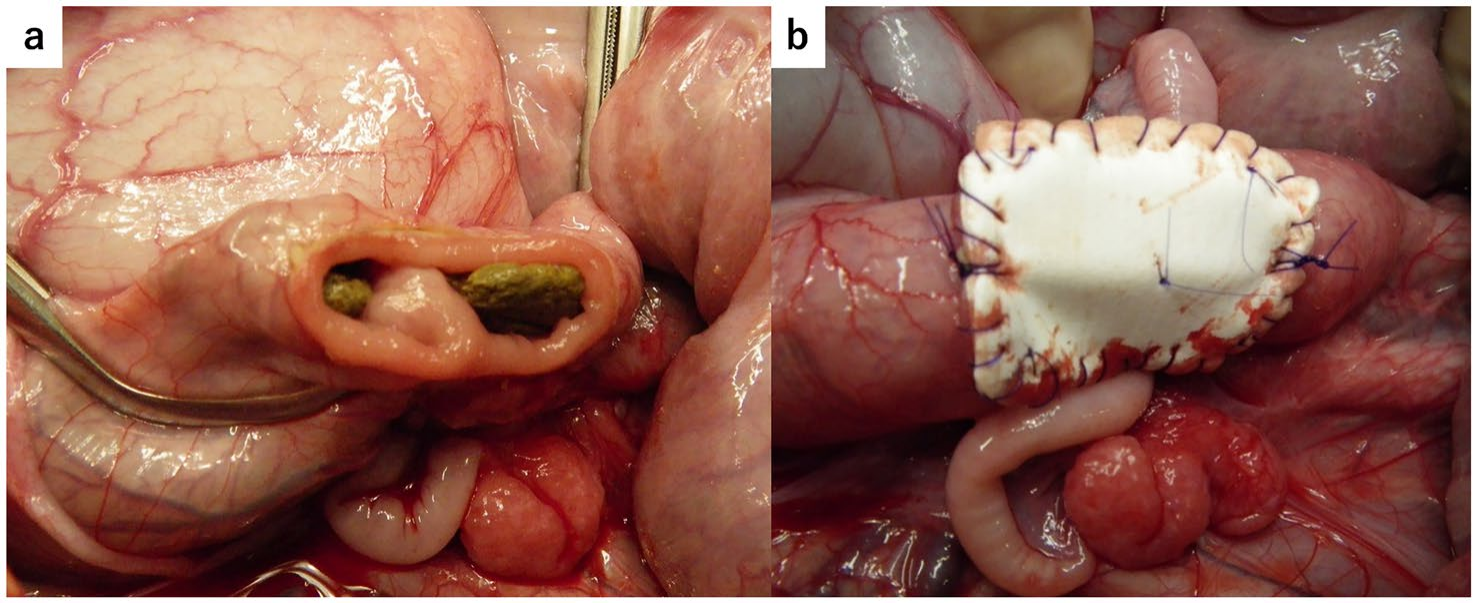
Macroscopic findings during transplantation. (a) A 2‐cm circular hole was created in the anterior wall of the sigmoid colon. Hard stool was observed inside the intestinal lumen. (b) A bioabsorbable polymer sheet was grafted onto the defect in the anterior wall of the sigmoid colon, followed by continuous suturing.

In this study, 10 mixed‐breed pigs (5 females and 5 males, 20–25 kg, 1–2 years old) were used. All pigs were housed at IVTec Labo (Narita, Japan) under standard husbandry conditions in individual pens. The animals were maintained in a controlled environment at a temperature of 20°C–24°C and a humidity of 40%–60%, with a 12‐h light/dark cycle. They had ad libitum access to commercial pig feed and water. No specific environmental enrichment was provided. All pigs were acclimatized for 14 days before the experiment in a controlled environment to reduce stress and ensure physiological stability. All pigs underwent 12 h of preoperative fasting. Anesthesia was induced with intramuscular ketamine (Ketalar; 20 mg/kg) and maintained with continuous intravenous infusion of propofol (Propofol) at a dosage rate of 0.2 mg/kg/min, followed by mechanical ventilation under general anesthesia. Just before the incision, cefazolin sodium (Cefamezin α; 1 g) was dissolved in 100 mL of normal saline and administered via intravenous drip. Each pig was placed in the supine position, and their abdomen was thoroughly disinfected with povidone iodine before upper midline laparotomy was performed to expose the sigmoid colon. A circular 2 × 2‐cm defect (approximately half the circumference) was created in the anterior wall of the sigmoid colon (Figure 2a). The artificial colon patch, designed to be slightly larger than the defect, with a diameter of 2.5 cm, was secured using a continuous suture with 4–0 PDS (Ethicon; Johnson & Johnson, Tokyo, Japan) to ensure complete closure (Figure 2b). Postoperatively, the pigs were fasted for 6 h, followed by solid feed introduction 12 h after surgery. No additional antibiotic treatment was administered postoperatively. Blood sampling and body weight assessments were performed at 1, 4, and 8 weeks after transplantation. The pigs were euthanized at 4 (*n* = 5) and 8 (*n* = 5) weeks after transplantation, and the sigmoid colon was excised and extracted for further evaluation.

Animals were monitored daily for signs of pain, distress, and health deterioration, including body weight loss (> 15%), abnormal behavior, reduced food or water intake, and respiratory distress. Humane euthanasia was planned for animals meeting these criteria; however, no animals met the criteria before the planned study endpoints.

### 2.3 Macroscopic and Histological Analyses

The transplanted site was examined macroscopically and histologically. Macroscopic evaluation included assessment of adhesion to the surrounding tissue and the presence or absence of stenosis and infection. For histological analysis, the excised specimens were fixed in formalin and stained with hematoxylin and eosin (H&E) and Elastica van Gieson (EVG). The regenerated mucosa and smooth muscle cells of the transplanted artificial colon were compared with those of the native colon to assess tissue regeneration.

### 2.4 Measurement of the Regenerated Colon Area

The areas of the artificial colon and regenerated colonic tissue were measured and compared. The artificial colon and the transplanted site were assumed to be circular in shape, and the area was calculated using the following formula: area = radius × radius × π. The contraction rate of the regenerated colon was then determined based on these measurements.

## 3 Results

### 3.1 Intraoperative Findings During BAPS Transplantation

In all cases, the colonic defect was successfully closed using the artificial colon without any complications. The procedure was performed with ease in all cases, and no intraoperative issues were encountered. No leakage of intestinal fluid from the transplanted site was observed (Figure 2b).

### 3.2 Postoperative Course

**FIGURE 3:**
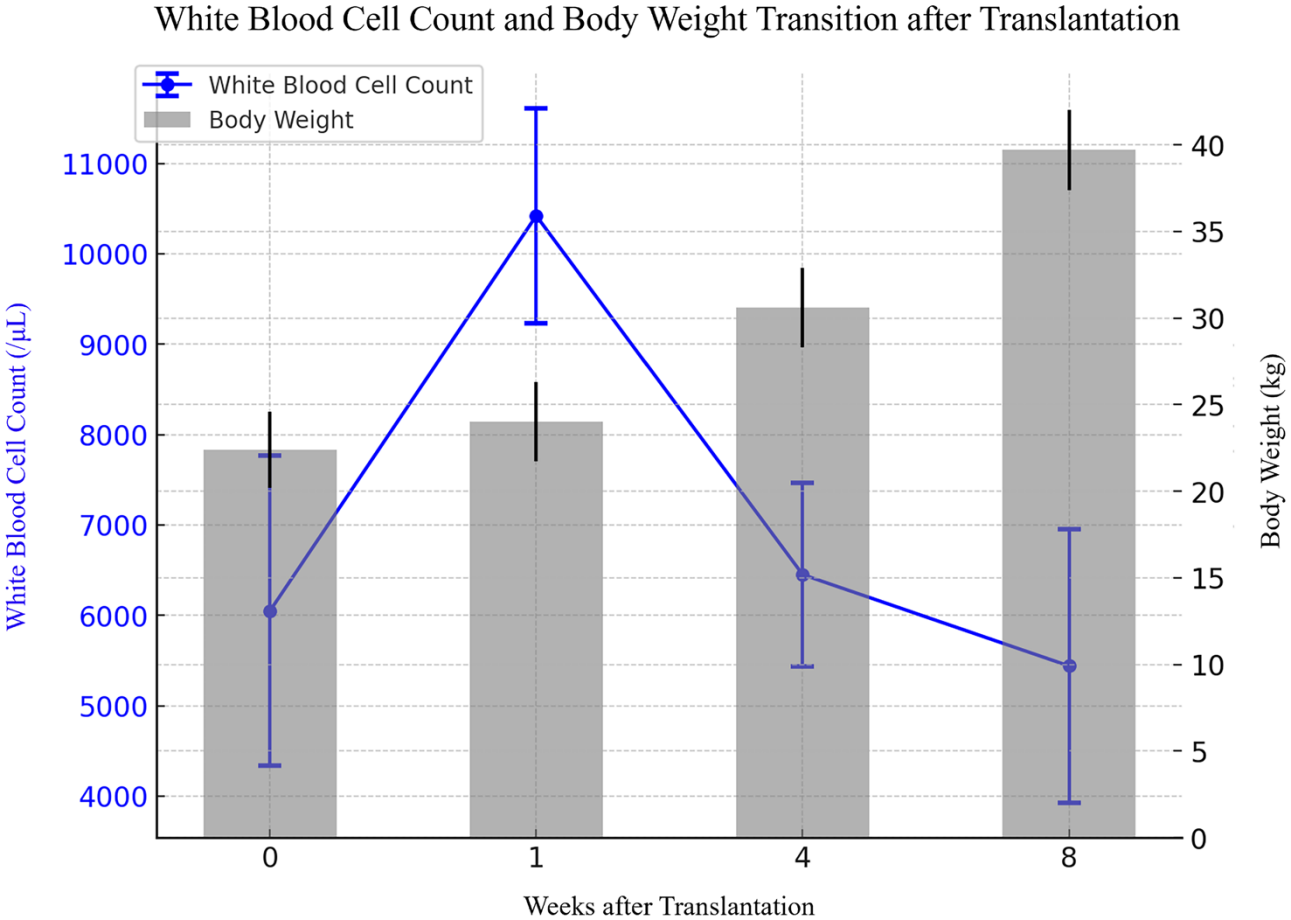
White blood cell count and body weight transition after transplantation. This table presents changes in the white blood cell (WBC) count and body weight after transplantation. The WBC count is shown as a blue line with error bars representing standard deviations, whereas body weight is represented by gray bars with standard deviations. The X‐axis represents the weeks after transplantation, with 0 indicating the pre‐transplant phase.

All pigs survived until they were euthanized. Throughout the postoperative period, no oral intake or defecation abnormalities were observed. Body weight increased steadily from 22.4 ± 2.2 kg preoperatively to 24.0 ± 2.3 kg at 1 week, 30.6 ± 2.3 kg at 4 weeks, and 39.7 ± 2.3 kg at 8 weeks after transplantation. The white blood cell counts were 6050 ± 1716/μL preoperatively, 10 420 ± 1187/μL at 1 week, 6450 ± 1018/μL at 4 weeks, and 5440 ± 1516/μL at 8 weeks after transplantation, indicating transient elevation at 1 week followed by subsequent improvement (Figure 3).

### 3.3 Macroscopic Findings

**FIGURE 4:**
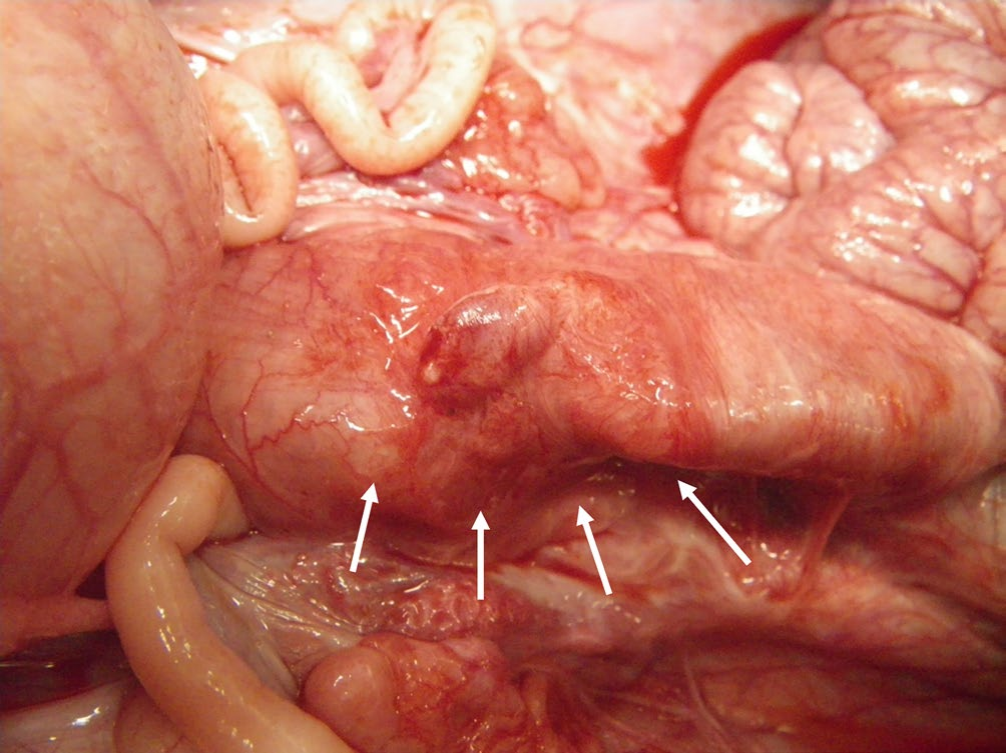
Macroscopic findings 4 weeks after transplantation. The bioabsorbable polymer sheet was absorbed and integrated with the surrounding sigmoid colon (arrows). Stenosis and deformation were not observed.

**FIGURE 5:**
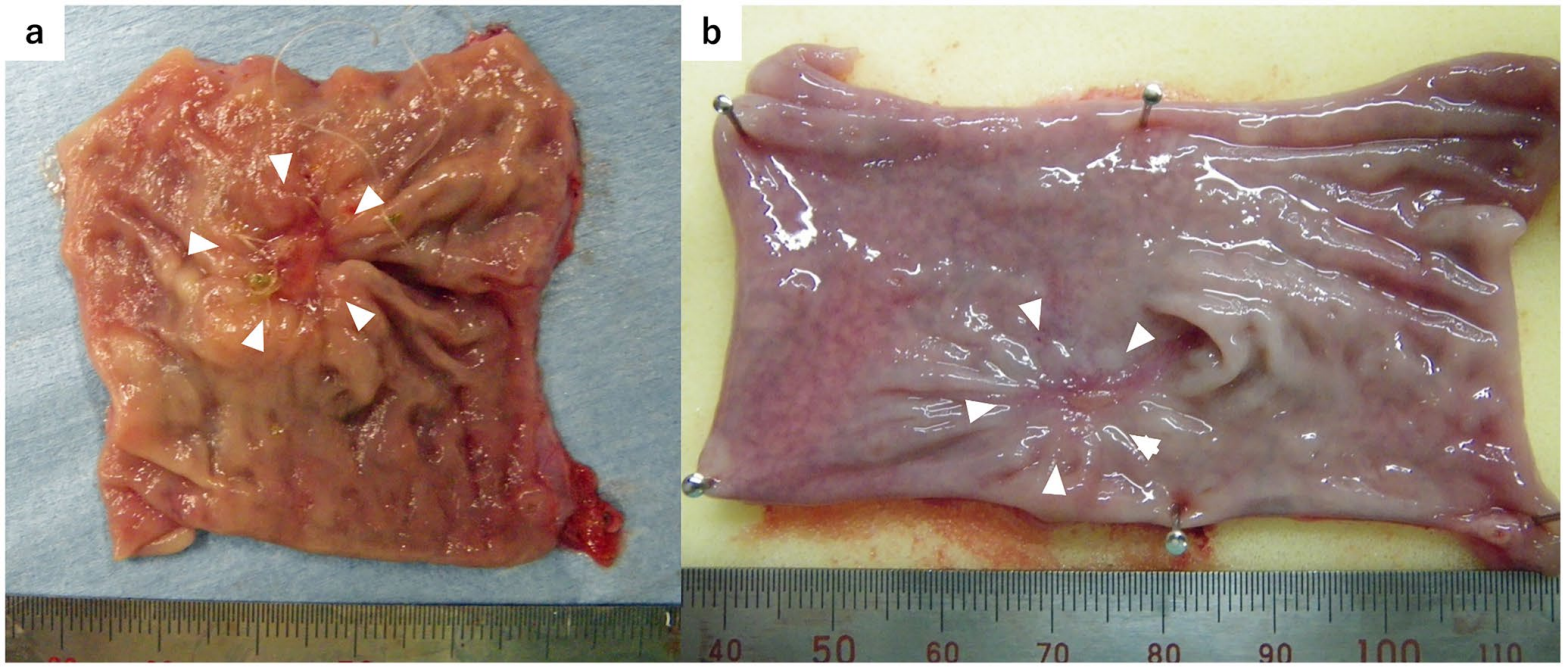
Lumen of the resected specimen at 4 and 8 weeks after transplantation. (a) Four weeks after transplantation, the transplanted site exhibited ulcer formation (white arrowheads), and the bioabsorbable polymer was completely absorbed. (b) Eight weeks after transplantation, the ulcer at the transplanted site had shrunk, which is the area indicated by the arrowhead, measured 1.94 × 1.85 cm (white arrowheads). The bioabsorbable polymer had disappeared.

At 4 weeks after transplantation, the BAPS had already disappeared macroscopically. No adhesions to the surrounding tissue were observed, and no signs of anastomotic leakage or infection at the transplantation site were observed (Figure 4). Observation of the resected specimen's lumen revealed the disappearance of the polymer at the transplantation site, along with ulcer formation (Figure 5a).

At 8 weeks after transplantation, no signs of intra‐abdominal infection were observed. Furthermore, no adhesions to the surrounding tissue were observed. In the resected specimen, the ulcer had reduced in size, and macroscopically, the transplantation site was indistinguishable from the surrounding tissue (Figure 5b).

### 3.4 Contraction Rate of the Regenerated Colon (8 Weeks After BAPS Transplantation)

The transplanted BAPS measured 2.5 × 2.5 cm with a total area of 4.9 cm^2^. Evaluation of the transplantation site in the resected specimens revealed a median diameter of 1.94 ± 0.03 × 1.85 ± 0.06 cm. The median transplantation site area in the resected specimens was 3.63 ± 0.14 cm^2^, indicating that the size of the transplanted part decreased by approximately 26%.

### 3.5 Histopathological Findings

**FIGURE 6:**
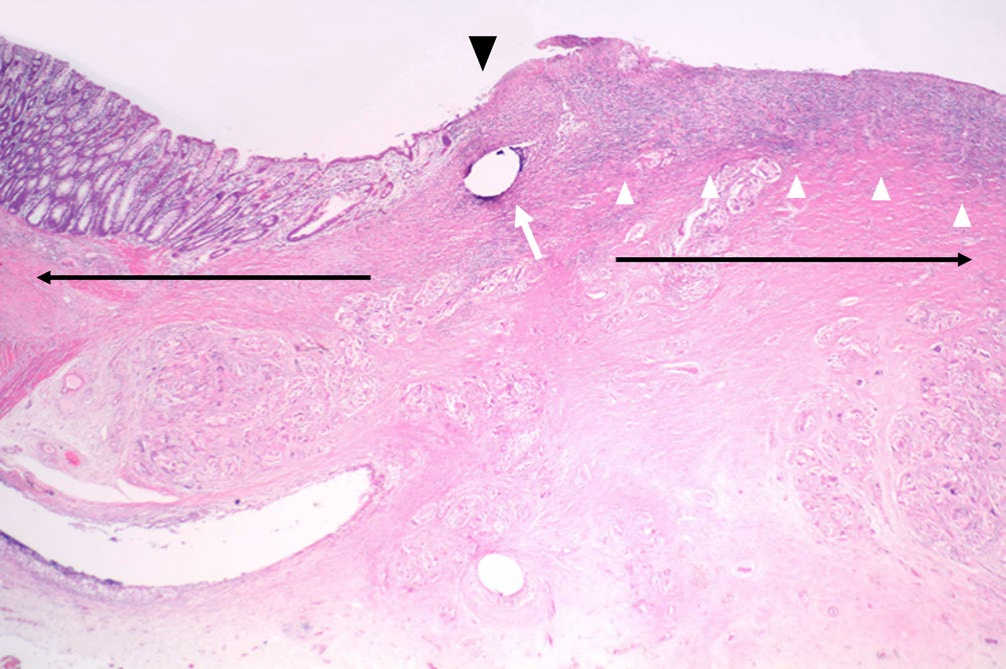
Pathological findings of the transplanted site 4 weeks after transplantation. Hematoxylin and eosin (H&E) staining revealed that the polymer had disappeared. Although epithelial regeneration was not observed, numerous immature cells were observed on the epithelial surface (white arrowheads). The white arrow indicates a suture hole, and the arrowhead marks the anastomotic site. The area to the right of the anastomotic site represents the BAPS graft site (horizontal rightward arrow), whereas the area to the left represents the native colon (horizontal leftward arrow) (magnification, ×20).

At 4 weeks after transplantation, H&E staining revealed the absence of polymer at the transplantation site. Although epithelial regeneration was not observed, numerous immature cells (infiltrated inflammatory cells) accumulated on the epithelial surface (Figure 6).

**FIGURE 7:**
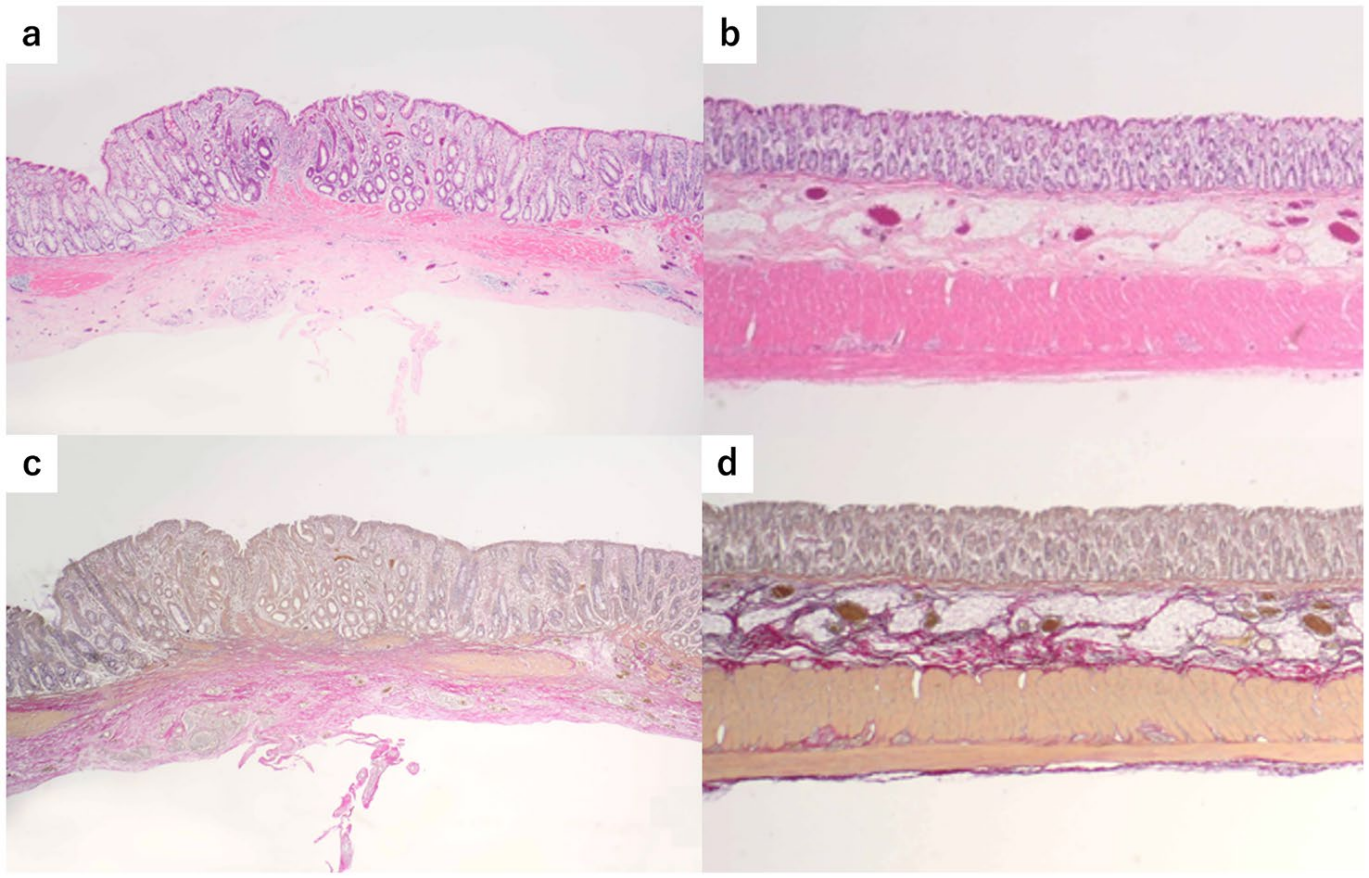
Pathological findings of the transplanted site and native sigmoid colon 8 weeks after transplantation. (a) Hematoxylin and eosin (H&E) staining revealed that the polymer had disappeared (magnification, ×40). (b) Histological findings of the native colonic wall stained with H&E (magnification, ×40). (c) Elastica van Gieson (EVG) staining revealed that the mucosa and muscularis mucosae exhibited fully regenerated structures similar to those of the native colonic wall; however, regeneration of the muscularis propria was incomplete compared with that of the native colonic wall (magnification, ×40). (d) Histological findings of the native colonic wall stained with EVG (magnification, ×40).

At 8 weeks after transplantation, H&E staining revealed no residual polymer. The epithelial and subepithelial structures closely resembled those of the native colon, although some irregularities were observed (Figure 7a,b). EVG staining revealed smooth muscle regeneration, reaching approximately one‐third of the native colon (Figure 7c,d).

## 4 Discussion

This study demonstrated the potential of a BAPS to regenerate approximately half the colonic circumference. The regenerated tissue had a structure similar to that of the native colon, and the colon functioned normally even after the BAPS was absorbed. To the best of our knowledge, no previous studies have reported colon regeneration while maintaining its normal structure. Therefore, this study is the first to report successful colon regeneration. However, this study was designed as an experimental model in which colonic perforations were immediately closed using BAPS, without reproducing the conditions of peritonitis. Therefore, caution is warranted when extrapolating these results to clinical practice. With further research, this approach might offer a less invasive and innovative treatment option for colonic perforation, serving as a simple solution comparable to “sealing a hole with a bandage.”

Recently, various studies on organ regeneration using organoids, ePTFE, and SIS have been conducted [8, 9, 10]. Although these methods hold promise as potential new treatment modalities, they still present significant challenges, and no clinically applicable regenerative therapy has yet been developed in the field of colorectal surgery [16]. ePTFE is a nonabsorbable material that is associated with a high risk of anastomotic leakage and intra‐abdominal abscess formation. SIS is a bio‐derived absorbable material that has not yet been approved for use in many countries when derived from pigs, and fatal zoonotic infections associated with the use of SIS have been reported. Organoids are limited to epithelial cell differentiation and proliferation, failing to achieve full colonic regeneration. Moreover, organoids require a prolonged period of functional integration, are available only in a few facilities, and currently lack sufficient clinical feasibility and readiness for practical application, restricting their widespread use. Furthermore, to effectively seal a perforation, a sheet‐like form with a certain degree of stiffness is required for clinical application. However, organoids lack this characteristic; thus, they are unlikely to be applicable for treatments aimed at closing perforations.

To develop a more practical approach for emergency use, we conducted a study using BAPS alone without cell seeding, with the aim of developing a simpler transplantation method. Using this approach, we previously demonstrated the feasibility of organ regeneration in the bile duct [11], esophageal wall [12], gastric wall [13], inferior vena cava [14], and portal vein [15]. In all cases, partial but successful organ regeneration was achieved while maintaining the structural integrity of the organ. The transplanted site demonstrated < 30% stenosis, indicating promising results. Building upon these findings, this study successfully exhibited the regeneration of colonic tissue with a structure closely resembling that of the native colon. Unlike animal‐derived materials, BAPS are not associated with risk of zoonotic infections and can be mass‐produced at a relatively low cost, making them easily accessible. Regarding safety, BAP is synthetic and composed of materials similar to those used in absorbable surgical sutures, which have been clinically validated through extensive surgical applications [17]. Considering its simplicity, biocompatibility, and effectiveness, the BAPS may have potential for application in the repair of colonic perforations. If endoscopic closure using the BAPS becomes feasible, it could provide a practical clinical option, particularly for iatrogenic perforations such as those following ESD. Currently, clip closure is the standard method for enabling conservative management of perforations after ESD or endoscopic mucosal resection. However, if the BAPS can be deployed endoscopically in a simple manner, it might enable the management of larger perforations and potentially reduce the need for surgery. While organoids provide a powerful model for epithelial function [18], they lack tissue‐resident immune cells, which are crucial for replicating organ‐level processes. In contrast, this study represents the first attempt to regenerate normal colonic tissue using a BAPS, and it holds promise as a model for regenerating normal colonic cells in the future. Therefore, this BAPS‐based approach may contribute to further advancements in regenerative medicine.

The sigmoid colon was selected as the transplantation site in our colonic regeneration experiments. The pressures in other organs we have studied previously were relatively low: 5–15 mmHg for the stomach, 6–12 mmHg for the small intestine, 10 mmHg for the bile duct, 5 mmHg for the inferior vena cava, and 10 mmHg for the portal vein. In contrast, the colonic pressure typically ranges from 6 to 49 mmHg but can rapidly increase above 100 mmHg during defecation [19]. Among the colonic segments, the sigmoid colon is known to experience the highest pressure, which is attributed to its narrow structure and the solidification of stool in this region. Furthermore, perforations caused by diverticulitis, ESD, and colorectal cancer most frequently occur in the sigmoid colon [1, 3, 4, 7]. Based on these factors, we determined that the sigmoid colon is the most appropriate transplantation site.

We evaluated the transplantation site at 4 and 8 weeks after transplantation. At 4 weeks after transplantation, we hypothesized that the transplanted site would be at its weakest. We determined whether a BAPS can withstand the high pressure of the sigmoid colon during this critical period. Although the BAPS is designed to undergo hydrolysis and degrade within 6–8 weeks, exposure to intestinal fluids can accelerate degradation, leading to earlier absorption. Our previous experiments confirmed that the BAPS had become fragile by 3 weeks after transplantation. Based on these findings, we anticipated that tissue weakness would peak at approximately 4 weeks after transplantation and designed our evaluation accordingly. At 8 weeks after transplantation, we assessed the degree of transplant site contraction. Our previous studies on the stomach and inferior vena cava demonstrated that transplant site stenosis plateaued at 8 weeks after transplantation and did not progress further [13, 14]. Thus, we considered this an appropriate time point to assess the final degree of stenosis. Our results were consistent with those of our previous studies on organ regeneration, showing that stenosis remained mild at < 30%. Unlike our previous experiments, this study was designed with an actual clinical application in mind. Considering the expected contraction rate, the BAPS was transplanted at approximately 150% of the size of the sigmoid colon defect. Therefore, intestinal deformation was successfully prevented, supporting its potential for future clinical application.

This study has two main limitations. The first limitation is the safety and efficacy of BAP transplantation in infected regions. To assess resistance to infection, the experiment was performed without postoperative antibiotic administration. No evident intra‐abdominal infections were observed, suggesting a certain degree of infection resistance. Furthermore, we have previously performed BAP transplantation experiments at infected sites, including the bile duct and anal fistula and have yielded favorable results [20, 21]. However, transplantation experiments under actual peritonitis conditions have not yet been conducted. In this experimental model, firm stool was observed in the sigmoid colon during transplantation (Figure 2a), indicating a higher localized risk of bacterial contamination. However, this does not replicate prolonged exposure or a true infectious setting, which represents a limitation of the model. Ideally, transplantation should be performed after a period following perforation, and this remains a subject for future investigation. Moreover, we have not performed in vitro experiments simulating peritonitis or infected conditions. Assessing the strength and functionality of the BAPS under such circumstances is essential for future clinical application and remains an important direction for further research. The second limitation is the lack of long‐term outcome evaluation. At 8 weeks after transplantation, histological analysis revealed that smooth muscle regeneration reached approximately one‐third of that in the native colon, and sufficient connective tissue and smooth muscle regeneration that could withstand the high pressure in the sigmoid colon was observed. Our previous studies have demonstrated that connective tissue and smooth muscle regeneration gradually increases over 1–2 years [14]. Considering that no stenosis, intestinal deformation, or functional impairment was observed in the midterm evaluation at 8 weeks, we speculate that long‐term outcomes would also be favorable. However, further experiments are necessary to verify this approach for clinical application.

## Author Contributions

**Junpei Takashima:** conceptualization, methodology, investigation, data curation, formal analysis, visualization, writing – original draft, writing – review and editing. **Mitsuo Miyazawa:** conceptualization, methodology, investigation, data curation, formal analysis, writing – original draft, writing – review and editing. **Masayasu Aikawa:** investigation, data curation, validation, writing – review and editing. **Daisuke Fujimoto:** investigation, data curation, validation, writing – review and editing. **Hirotoshi Kobayashi:** supervision, project administration, writing – review and editing, validation.

## Ethics Statement

This study was approved by the Animal Experimentation Ethics Committee of IVTec Labo (IVT24‐59), and the animals were treated according to the National Institutes of Health Guide for the Care and Use of Laboratory Animals. Animal experiments were performed following the Animal Research: Reporting of In Vivo Experiments guidelines to ensure methodological rigor and ethical compliance.

## Consent

The authors have nothing to report.

## Conflicts of Interest

The authors declare no conflicts of interest.

## Data Availability

All data generated or analyzed during this study are included in this published article.
